# Increased Likelihood of Pregnancy Using an App-Connected Ovulation Test System: A Randomized Controlled Trial

**DOI:** 10.1089/jwh.2019.7850

**Published:** 2020-01-13

**Authors:** Sarah Johnson, Joseph B. Stanford, Graham Warren, Sharon Bond, Sharon Bench-Capon, Michael J. Zinaman

**Affiliations:** ^1^ Clinical Research Department, SPD Development Company Ltd., Bedford, United Kingdom.; ^2^ Division of Public Health, Department of Family and Preventive Medicine, University of Utah, Salt Lake City, Utah.; ^3^ Department of Obstetrics and Gynecology, Jacobi Medical Center, Albert Einstein College of Medicine, Bronx, New York.

**Keywords:** Clearblue Connected Ovulation Test System, conception rates, luteinizing hormone, ovulation testing, pregnancy

## Abstract

***Background:*** Women trying to conceive are increasingly using fertility-tracking software applications to time intercourse. This study evaluated the difference in conception rates between women trying to conceive using an application-connected ovulation test system, which measures urinary luteinizing hormone and an estrogen metabolite, versus those trying without using ovulation testing.

***Materials and Methods:*** This home-based study involved 844 volunteers aged 18–40 years seeking to conceive. Volunteers randomized to the test arm were required to use the test system for the duration of the study while those randomized to the control arm were instructed not to use ovulation testing. Pregnancy rate differences across one and two cycles between the two groups were examined.

***Results:*** Volunteers in the test (*n* = 382) and control arms (*n* = 403) had similar baseline demographics. The proportion of women pregnant after one cycle was significantly greater in the test arm (25.4%) compared with the control arm (14.7%; *p* < 0.001). After two cycles, there continued to be a greater proportion of women pregnant in the test arm compared with the control arm (36.2% vs. 28.6%; *p* = 0.026). In the test arm, volunteers had intercourse less frequently per cycle compared with those not using ovulation testing (9 [range: 1–60] vs. 10 [range: 1–50]; *p* = 0.027), but were more likely to target intercourse to a particular part of their cycle compared with those not using ovulation testing (88.5% vs. 57.8%; *p* < 0.001).

***Conclusion:*** Using the test system to time intercourse within the fertile window increases the likelihood of conceiving within two menstrual cycles.

## Introduction

Women are increasingly choosing to postpone pregnancy until they reach a period in life when raising children is consistent with their career paths or life goals.^1^ This often means that once they do decide to start trying to conceive they often wish to become pregnant quickly, leading to frustration if they fail to do so.^2–4^ Conception is most likely to occur when intercourse takes place within the fertile window, which begins ∼3–5 days before ovulation (dependent on the lifespan of the sperm) until approximately the day of ovulation.^5,6^ Although the timing of sexual intercourse greatly influences the chances of becoming pregnant, many women seeking to conceive appear to have an inaccurate perception of their ovulatory pattern.^7,8^

There is a high degree of intra- and inter-individual variation, both in the length of the menstrual cycle and the relative day of ovulation, which means the fertile window can vary considerably.^9,10^ Even for women with an average cycle length of 28 days, ovulation can occur as early as day 11 and as late as day 20 in the cycle.^10^

For women desiring pregnancy, timing of intercourse to coincide with a woman's most fertile time can be facilitated by monitoring key fertility hormones, which can be conveniently performed in a home-based setting using urinary ovulation testing.^11^ Tracking the luteinizing hormone (LH) surge in urine has been shown to be a highly reliable indicator of impending ovulation.^11–13^ Ovulation typically occurs ∼24 hours after the onset of the LH surge and will not occur in its absence.^12,14,15^ Estradiol is another key hormone that can be used to assess relative fertility. Levels of a principal urinary estradiol metabolite, estrone-3-glucuronide (E3G), have been observed to rise substantially ∼3 days before ovulation until about 5 days postovulation, which can make it an excellent predictor of the onset of the full fertile window.^12,14^

A randomized controlled study found that measuring LH and E3G across the entire menstrual cycle using a fertility monitor was associated with an increased rate of conception.^16^ There is also some evidence to suggest that use of digital home ovulation tests may increase the chances of conception^17^; however, to date no randomized controlled studies to examine pregnancy rates when using home ovulation tests for urine LH and/or estrogen metabolites have been published.

Fertility-tracking software applications (apps) designed for use on smartphones and similar devices are increasingly being used by women trying to conceive to time intercourse.^18^ Most fertility apps make predictions solely generated from user data, such as the date of the last menstrual period and cycle length.^19^ However, using calendar methods alone to detect the fertile window and ovulation has been shown to lack predictive accuracy.^10,19,20^ One study found that the probability of correctly identifying a woman's day of ovulation using cycle-tracking apps was no better than 21%.^10^ Therefore, a method of identifying the fertile window that combines the accuracy of an ovulation test with the convenience of an app could be of benefit to women seeking to conceive.

The Clearblue Connected Ovulation Test System (Swiss Precision Diagnostics [SPD] GmbH, Geneva, Switzerland) is designed for home use by women and is able to accurately predict the fertile window by tracking elevations in levels of LH and E3G, which precede ovulation.^21^ The test system is able to connect via Bluetooth to the user's smartphone, where the app records information relating to the woman's menstrual cycle and uses this information to determine when to conduct the urine tests. Through the urine hormone measurement, the test reports one of three levels of fertility: low when hormone levels are at baseline, high when the monitor detects increasing E3G levels, and peak fertility upon detection of the LH surge.

The objective of this study was to establish whether women trying to conceive using the Clearblue Connected Ovulation Test System had a higher pregnancy rate compared with those trying to conceive without using ovulation tests.

## Materials and Methods

This was an open, home-based, randomized control trial (clinical trial number NCT03424590) of women aged 18–40 years who were actively trying to conceive. Volunteers were screened for inclusion in the study via an online survey; if eligible, they were informed of the study details and, if still interested in participation, completed the volunteer information form and consent form. Study inclusion criteria included women who were actively trying to conceive and willing to use their own smartphone, disclose their pregnancy status, and provide urine samples, and who had at least two regular consecutive cycles since last pregnancy/miscarriage and had a smartphone compatible with the system. Women were excluded if they were employed or had a relative employed by SPD or parent company of SPD, had a reason that they should not get pregnant, had been trying to conceive for >6 months (criterion was <3 months for women aged ≥35 years), had previously been diagnosed with polycystic ovary syndrome, or were using any infertility medications. Women were also excluded if they were currently pregnant, breastfeeding, peri-menopausal, postmenopausal, or using a form of contraception. A total of 1000 women were recruited from England, Wales, and Scotland to conduct the study in their own home, of whom 844 were randomized and entered the first cycle of the study.

**FIG. 1.**
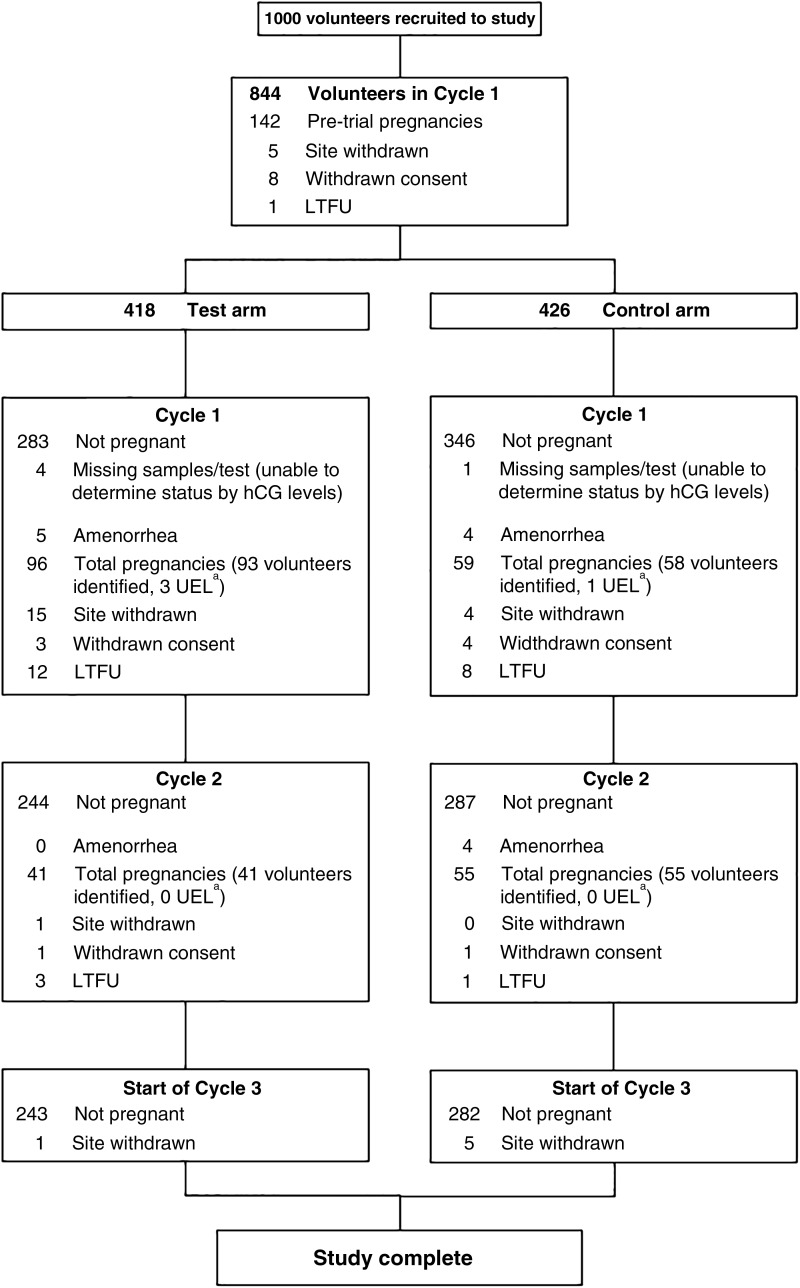
Flowchart of volunteers through the study. Of the 1000 volunteers recruited, 128 did not enter randomization (118 pregnant before study start, 7 withdrew consent, 2 site withdrawn, 1 LTFU), resulting in 436 volunteers randomized to each arm of the study. Pretrial pregnancy means the volunteer was recruited to study but discovered pregnancy before beginning the study. Site withdrawn was due to noncompliance with protocol. In the test group, there were nine pretrial pregnancies, three site withdrawals, and one withdrawn consent before the beginning of the study. In the control group, there were six pretrial pregnancies before the beginning of the study. ^a^These volunteers had pregnancies as identified by hCG being present in urine samples but were unaware that they had conceived so progressed into cycle 2. hCG, human chorionic gonadotropin; LTFU, lost to follow-up; UEL, unidentified early loss.

For an estimated test pregnancy proportion at the primary endpoint of 25% and an odds ratio of 1.9, the required sample size with a significance level of 5% and power 90% was calculated to be 346 per arm. To ensure that there were enough volunteers by the end of the study, over 400 volunteers per arm were randomized. Participant flow for the study is shown in Figure 1.

Once consent had been received, volunteers were assigned a unique volunteer number, then randomized 1:1 into the test or control arm. Randomization was stratified by the age of the volunteers, with two cohorts (<35 and ≥35 years of age). Block randomization was used to ensure a balance of numbers in each group.

Volunteers assigned to the test group were required to use the test system in their homes according to instructions provided for up to two complete menstrual cycles. Women assigned to the control group were required to continue trying to conceive but were told not to use any ovulation tests for the duration of the study. Women in the control group who reported urine ovulation test use during the study were withdrawn (*n* = 4, all not pregnant at end of cycle 2). All participants received the same monetary reimbursement for their participation at study completion, regardless of how many cycles for which they participated or their pregnancy status. All participants in the control group received a 2-month supply of home ovulation tests at the end of the study, again regardless of pregnancy status or number of cycles completed. This information was provided to volunteers at study outset to prevent behavioral bias due to differential reimbursement.

The efficacy endpoints were the difference in the proportion pregnant across one cycle and two cycles of use between volunteers trying to conceive using Clearblue Connected Ovulation Test System and no intervention. Other endpoints included intercourse frequency and timing, and the quantitative assessment of methods used to assist with conception. Intercourse data were collected retrospectively, because compulsory diary recording of behaviors would introduce an additional intervention, but we did not prohibit volunteers from recording on their own apps/diaries if this was their normal behavior.

Volunteers were provided with Clearblue Digital Early Detection Pregnancy Tests (SPD GmbH, Geneva, Switzerland), urine sample pots, and a form to record menses to determine pregnancy status at the end of each cycle. Volunteers were asked to collect a urine sample and also to conduct a pregnancy test on specified test days. The sample and used test were immediately returned to the study site by post. The first test date was the day of the missed period, calculated from cycle history provided by the volunteer using the following formula: last menstrual period date + average cycle length +1 day. The testing dates were calculated by the study site and provided to the volunteer. Testing was conducted irrespective of whether menses had occurred.

If results were negative and their period had not started, volunteers were asked to collect a new sample and test again 5 days after the previous test. If they had negative results and their period had not started before the last test date provided (21 days after period would have been expected), they were classed as having amenorrhea and exited from the study in the cycle in which amenorrhea occurred. Amenorrheic volunteers were classed as not pregnant in the efficacy endpoint analyses.

On receipt at the study site, the digital home pregnancy test results were confirmed by inspection of device LCD screen (or download of digital device if screen was blank). Urine samples were stored under previously validated conditions: 4°C until analysis or at −80°C if the analysis could not be performed within 2 weeks of receipt. Urinary human chorionic gonadotropin (hCG) was quantified in all samples using an AutoDELFIA™ (Perkin Elmer, Waltham, MA) assay.

The total number of pregnancies was determined by confirming the presence of hCG in the volunteer's urine by quantitative measurement of the urinary hCG concentration in the returned urine sample, which was available for all completed volunteers.

The efficacy endpoints for each cycle were calculated using a Fisher's exact test 2 × 2 contingency table. The proportion of pregnant and not-pregnant volunteers in each arm after two cycles was calculated and the odds ratio was calculated based on the formula: [Pt/(1−Pt)]/[Pc/(1−Pc)] where the proportion pregnant volunteers in the control group = Pc and the proportion of pregnant volunteers in the test group = Pt. A Kaplan–Meier analysis was carried out to estimate the time-to-conception distributions in the two arms. The study was approved by the SPD Ethics Committee on January 17th 2018 (protocol-0987) and all procedures were conducted in accordance with relevant regulations and guidelines.

## Results

Volunteers were randomized 1:1 into test and control arms (*n* = 436 per group), but a number of randomized volunteers exited the study before commencement, mostly due to becoming pregnant before entering their study cycle. This resulted in 423 volunteers beginning the study in the test group and 430 in the control group (Fig. 1). Completed volunteers in the test arm (*n* = 382) and the control arm (*n* = 403) had similar baseline demographics (Table 1). The proportion of total pregnancies after one cycle was significantly greater in volunteers using the test system (25.4%) compared with volunteers not using any ovulation testing (14.7%; *p* < 0.001), odds ratio of 2.0 (95% confidence interval [CI]: 1.4–2.8).

**Table 1.** Baseline Demographics

Demographics	Test arm	Control arm
Age, years (range)	30 (18–40)	30 (18–40)
Self-reported average cycle length, days (range)	28 (20–45)	28 (21–42)
Self-reported shortest cycle length, days (range)	27 (18–42)	27 (14–41)
Self-reported longest cycle length, days (range)	30 (21–47)	30 (22–56)
Weight, kg	72.75 ± 16.74	72.43 ± 16.18
Height, m	1.66 ± 0.07	1.65 ± 0.07
Body mass index, kg/m^2^	26.43 ± 5.65	26.48 ± 5.68
Smoking status		
Current	14 (3.7%)	17 (4.2%)
Previous	92 (24.1%)	109 (27.0%)
Never	276 (72.3%)	277 (68.7%)
Taking folic acid		
Yes	307 (80.4%)	306 (75.9%)
No	74 (19.4%)	95 (23.6%)
Don't know	1 (0.3%)	2 (0.5%)
Used contraception in last 12 months		
Yes	276 (72.3%)	275 (68.2%)
No	106 (27.7%)	128 (31.8%)
Months since stopping contraception (range)	4 (1–12)	4 (1–11)

Median (range) or mean ± standard deviation or count (%) of total.

Across two cycles cumulatively, a greater proportion of pregnancies was seen among volunteers using the Connected Ovulation Test System (36.2%) compared with volunteers who did not use any ovulation tests (28.6%; *p* = 0.026) (Table 2), giving an odds ratio of 1.4 (95% CI: 1.01–1.9). Kaplan–Meier survival analysis also indicated that the pregnancy rate over two cycles was significantly greater when using the test system compared with not using an ovulation test (*p* = 0.015). An intention-to-treat analysis, which considered the lost to follow-up volunteers as not pregnant, yielded equivalent results; one cycle odds ratio 2.0 (95% CI: 1.4–2.8), two cycle odds ratio 1.4 (95% CI: 1.03–1.9).

**Table 2.** Pregnancy Proportions and Odds Ratios for Test Versus Control Groups

	Pregnancy rate (95% CI)		Odds ratio (95% CI)	Fisher's exact test* p *value
	Test group	Control group		
1 cycle total pregnancies	25.4% (21.1–30.1%)	14.7% (11.4–18.5%)	2.0 (1.4–2.8)	<0.001
2 cycle total pregnancies	36.2% (31.3–41.3%)	28.6% (24.2–33.4%)	1.4 (1.01–1.9)	0.026

CI, confidence interval.

Based on the end-of-study follow-up questionnaire, volunteers using the test system were found to have intercourse slightly less frequently per cycle compared with volunteers who did not use any ovulation testing (9 vs. 10; *p* = 0.027) (Table 3). Unexpectedly, despite a lower average frequency of intercourse, a greater proportion of volunteers using the test system perceived that they were having intercourse more frequently (compared with before the study) than did volunteers not using ovulation testing (30.4% vs. 21.1%; *p* = 0.004).

**Table 3.** Self-Reported Retrospectively Collected Intercourse Frequency

Intercourse frequency per cycle (range)	Test arm		Control arm		p
	9	(1–60)	10	(1–50)	0.027
More or less frequent during the study period, compared to previous intercourse frequency?					
More frequent	116	30.4%	85	21.1%	0.004
About the same	242	63.4%	299	74.2%	
Less frequent	22	5.8%	14	3.5%	
Not sure	2	0.5%	5	1.2%	
Did you focus intercourse to any particular part of the cycle?					
Yes	338	88.5%	233	57.8%	<0.001
No	44	11.5%	170	42.2%	

A large proportion of volunteers using the test system reported that they targeted intercourse to a particular part of their cycle compared with those not using ovulation testing (88.5% vs. 57.8%; *p* < 0.001). Interestingly, the higher the number of intercourse acts, the lower the likelihood of conception; odds ratio for pregnancy 5.9 for ≤5 acts, 4.0 for 6–10, 2.6 for 11–15, and 1.0 for 16–20 versus >20 acts. No volunteers from either group reported use of contraception during the study.

At the end of the study, both groups were asked whether they used specific methods to identify the fertile window (Table 4). The most popular of these methods was found to be the use of apps, followed by cervical mucus testing and sex drive, with the least popular being mid-cycle spotting and wearable devices. Use of nonstudy cycle tracking apps was not associated with likelihood of pregnancy.

**Table 4.** Self-Reported Use of Methods Other than Clearblue Connected Ovulation Test System to Identify Fertile Window

Method: Did you use any of the methods below to identify your fertile time while on the study?	Test arm	Control arm
	% selected	% selected
App	N/A	40.0
Cervical mucus	32.7	35.2
Sex drive	13.1	15.6
Body basal temperature	7.6	6.5
Moods	7.3	6.2
Cervical position	5.0	3.2
Mid-cycle spotting	0.8	0.5
Wearable device	0.8	0.5
None	19.4	39.0
Other	1.0	4.5

N/A for app use in test arm because it was not possible to determine whether the response from volunteers in the test arm referred to a study app, or an additional nonstudy app.

App, application; N/A, not applicable.

## Discussion

This study found that, for women using a home ovulation test with connected app, the odds of becoming pregnant were twice those for women not using ovulation testing in the first cycle of use. Similarly, after two cycles, a greater proportion of women using the test system became pregnant compared with women not using ovulation testing (36.2% vs. 28.6%; *p* = 0.026).

This is the first randomized controlled study conducted to examine the efficacy of a home urine ovulation test system paired with an app. Women assigned to the connected ovulation test that detects the wider fertile window were more likely to conceive, particularly in first cycle of use. Therefore, use of Clearblue Connected Ovulation Test System may be particularly beneficial for couples seeking to conceive quickly.

There is now a plethora of products available to women seeking to conceive naturally. Fertility tracking apps are becoming increasingly popular among women seeking to conceive.^18^ Many of these fertility apps base their predictions for the fertile window solely on menstrual cycle characteristics, a method that has been associated with a lower level of predictive accuracy compared with other fertility monitoring methods such as urinary ovulation testing.^10,22,23^ These apps also assume each woman's fertile period is the same length—typically 6 days—ignoring the evidence that the length of the fertile window differs between women.^24^ It is possible that inaccurate information on the fertile period could be detrimental to chances of conceiving, particularly if intercourse frequency is low.^25^ It is interesting that many volunteers in the control group were using fertility apps during the study but they did not increase conception chances. However, many cycle apps may not be helpful for accurately timing intercourse.^10,20^

Analysis of the end-of-study questionnaire revealed that despite a higher average frequency of intercourse in the control group, use of the test system was associated with a higher conception rate. This supports the hypothesis that correct timing of intercourse increases likelihood of conception.^22,26^

For women in the early stages of trying to conceive, information on the appropriate timing of intercourse is simple and effective advice that can be easily provided. The information collected by the app-connected ovulation test system could also be used to assist with patient management; for example, objective evidence of failure to conceive following 6 months of intercourse timed to ovulation as predicted by the LH surge may suggest the need for an investigation for male factor issues. Three consecutive cycles without an LH surge could prompt investigation for ovulatory issues.

Other cycle-monitoring techniques that utilize urinary hormone testing have demonstrated similar increases in pregnancy rates. Use of the Clearblue Easy Fertility Monitor (SPD GmbH), which also measures E3G and LH to predict the fertile period, was found to increase the likelihood of conception during the first two cycles of use in women who had been trying to conceive for up to 2 years.^16^ The connected system tested here is similar to the fertility monitor in that it measures the same hormones to identify the fertile phase. However, it has a very different usability profile, as the connected app is able to give more detailed guidance to the user regarding how and when to test and what the results mean; it also provides reminders. Women tend to have their smartphones to hand at all times, meaning that fertility information is also much more accessible. However, there is very little research regarding the value of having immediate access to health information. While there are very good-quality resources available, there is also a considerable amount of poor-quality information and this could hinder, rather than help, women seeking to become pregnant. Further user-centered research is desperately required on app-based technologies.

Randomized, controlled studies provide robust evidence of the efficacy of an intervention, providing no bias has been introduced. Therefore, considerable care was taken to ensure test and control groups were treated identically, except for use of the connected ovulation test system. It was also important not to accidentally introduce additional interventions. Observational diaries are frequently used as a tool to accurately assess behavior.^27^ However, studies have reported evidence of systematic behavior change with observational diary use, such that diary recording can be considered an intervention.^28^

This study was designed to be as unobtrusive as possible and did not require volunteers to keep a daily intercourse diary or perform any other continuous record-keeping. This was to mimic true conception behavior as closely as possible and provide a realistic estimate of what women at home could expect. Consequently, retrospective intercourse data may be less reliable than data collected prospectively. This may explain why our study found a mismatch between reported intercourse frequency and perception as to whether intercourse was more frequent than at baseline—it is possible that women perceive an increase in frequency when intercourse is targeted across a short time period.

An important finding was that the probability of conception was highest for those reporting least intercourse. The majority of volunteers claimed not to alter their intercourse frequency during the study. Therefore, for couples having intercourse 10 or more times per month, it is highly likely that they will have had intercourse during the fertile period before entering the study, so the reason for failing to conceive is likely due to a cause other than mistiming of intercourse; for example, male factor. Therefore, using a home ovulation test to identify the optimum time for intercourse is not likely to be of benefit to these couples. Conversely, given the extremely large uplift in pregnancy rates in those having intercourse <5 times per month, timing appears critical for these couples to help achieve pregnancy. Couples having very frequent intercourse without pregnancy success could be advised to seek health care professional advice sooner.

A limitation of this study was that use of the test system was only investigated across two menstrual cycles. Therefore, this study does not provide evidence of cumulative pregnancies over longer-term use.

This study found that use of the test system comprising urine testing of E3G and LH paired with an app was associated with a significantly higher rate of pregnancy after one and two cycles compared with no use of ovulation tests. This indicates that using the test system to time intercourse within the fertile window increases the likelihood of conceiving for women who have recently started trying to conceive.
